# Circular RNAs in gynecological disease: promising biomarkers and diagnostic targets

**DOI:** 10.1042/BSR20181641

**Published:** 2019-05-17

**Authors:** Jie Huang, Qin Zhou, Yunyun Li

**Affiliations:** 1 Department of Gynecology and Obstetrics, The First Affiliated Hospital of Chongqing Medical University, Chongqing 400016, P.R. China; 2 Department of Gynecology and Obstetrics, The Yongchuan Hospital of Chongqing Medical University, Chongqing 402160, P.R. China

**Keywords:** cervical cancer, Circular RNAs, gynecological disease, ovarian disease

## Abstract

Circular RNAs (circRNAs) are a category of RNA molecules with covalently closed circles lacking both a 5′ cap and a 3′ tail. In recent years, circRNAs have attracted much attention and become a research hotspot of the RNA field following miRNAs and lncRNAs. CircRNAs exhibit tissue specificity, structural stability, and evolutionary conservation. Although the biological effects of circRNAs are still underestimated, many studies have shown that circRNAs have functions including regulation of transcription, translation into proteins and miRNA sponges. In this review, we briefly described the biogenesis and function of circRNAs and present circular transcripts in gynecological disease.

## Background

Circular RNAs (circRNAs) are the newest addition to the ever-growing world of RNA molecules. Unlike classical RNA formation, circRNAs are constructed from linear pre-messenger RNAs (pre-mRNAs) through a process termed ‘back-splicing’ and give rise to a covalent closed loop structure without the 5′ and 3′ ends. With the widespread application of gene sequencing technology and bioinformatics methods, many studies have shown that circRNAs are abnormally expressed in many malignancies such as breast cancer, lung cancer, colorectal cancer, and osteosarcoma [1,2]. For example, Zhang et al. proposed that circRNAs might be involved in the tumorigenesis and regulation of related signaling pathways in breast cancer cells, as they found that the abundance of circRNAs expressed by genes encoding protein kinases in the mammary gland, which were significantly higher than other coding genes [3].

In the field of gynecology, reports on circRNAs mainly focus on screening and expression patterns. These studies revealed the role of circRNA in gynecological disease.

## Discovery of circRNAs

As early as 1979, circRNAs were first found in the eukaryotic cytoplasm through electron microscopy [4]. However, due to their low abundance and unconventional structural features, coupled with the limitations of detection techniques at that time, circRNAs were once considered to be byproducts of abnormal splicing and thus were ignored for a long time [5]. In 1991, when Nigro et al. studied the expression of tumor suppressor genes, they observed that the exons of tumor suppressor DCC were different from that of its gene sequence. The upstream 5′ exons moved downstream of 3′ exons, were bound in a nonsequential manner, and produced a circular isoform of DCC. They called this new RNA transcript scrambled exons [6]. The studies of circRNAs were only sporadically reported in the following 20 years. The breakthrough came in 2012, with the advancement of high-throughput sequencing technologies and bioinformatics, Salzman et al. underlined the abundance of circRNA species in mammalian cells, discovering, and annotating thousands of circular transcripts. It has been estimated that approximately 100,000 circRNAs are expressed in human cells [7].

## Biogenesis of circRNAs

CircRNAs are widely found in archaea, bacteria, viruses, and human cells, mainly in the cytoplasm of eukaryotes, except that the nucleus contains a small amount of circRNAs derived from introns [8,9]. Exons easily circularize to form circRNAs; in addition, introns, lncRNAs, antisense transcripts, and intergenic regions are also susceptible to form circRNAs. According to the original gene location, circRNAs can be named exon circRNA [10], intron circRNA [11], exon-intron circRNA [12], and tRNA circular RNA (tricRNA) [13]. CircRNAs originating from coding regions can be combined by two or three pre-mRNA exons that are larger than the average length, sometimes including introns [10]. Intron circRNAs are usually formed by direct splicing of intron sequences and then forming a lariat structure [8]. TricRNAs are usually produced during tRNA processing.

Back-splicing is a well-established way to form a circular transcript, in which the head-to-tail splice junctions are joined together, and some mechanisms have been reported to induce back-splicing. One method is called exon skipping. The pre-mRNA is spliced to form two transcripts: one transcript is a lariat which contains exons and introns by jumping splicing, and the other transcript is the mRNA in which one or more exons are missing. The exon lariat is spliced again to form a circRNA and an intron lariat [14]. Another method circRNAs are produced is by intron paring. The difference is that two introns flanking the exon or exons of the nascent circRNA have a complementary structure. The flanking intron pairing brings the splicing sites close to each other, creating a secondary structure that makes circularization possible [15]. In addition, circRNAs can be directly regulated by RNA binding proteins (RBPs). The most classical example of RBPs is the formation of the circRNA cSMARCA5, in which the RBP Quaking (QKI) binds to each side of the flanking introns and dimerize, creating a bridge between them, eventually leading to the formation of circular transcripts [16].

In addition to the above reports, there are other ways to form circular transcripts. In archaea, circRNAs are mainly isolated from precursor RNA by endonuclease and then ligated into circRNA by RNA ligase. The viroid genome, satellite RNA, and the human hepatitis delta virus genome are naturally occurring circular RNAs. Genes are duplicated from a rolling circle mechanism that uses one or two polar circular templates for repeated transcription and resulting in oligomeric RNA. These oligo RNAs are then cleaved into monomeric RNA and ligated into circRNAs [8].

The biogenesis of circRNAs has not yet been fully deciphered, and the mechanism described above is only a limited point of view. Understanding the biogenesis of circRNAs will enable the development of computing tools to predict genome landscapes susceptible to circularization.

## Features of circRNAs

Compared with other kinds of RNAs, conservation, stability, and specificity are the three major features of circRNAs. First, circRNAs exhibit strong tissue-, time-, and disease-specificity, with an expression level that is ten times higher than that of their linear isomers. In 2015, the journal of Cell Research reported an increase in 257 and 67 circRNAs lost in the exosomes of colorectal cancer patients compared with normal people. These different circRNAs could be used as specific markers for colorectal cancer [17]. Second, the circular structure of circRNAs is resistant to degradation by RNase, so circRNAs exhibit increased structural stability compared with linear RNA. The half-life of circRNAs is approximately 48 h, which is two to four times longer than that of homologous mRNA [10]. Finally, the conservation of circRNAs is characterized by the similarity of circRNA sequences amongst the same tissues between different species. A study by Salzman et al. mentioned that there were hundreds of similar transcripts in the brains of mice and humans, but no details were provided about the basic structural similarities of these molecules [7]. Rybak-Wolf et al. found 4522 of the 15,849 circRNAs in human and mouse neurones that were highly conserved between the two species [18]. The gene sequence encoding circRNAs between different species overlap. For example, human and murine circRNA genes overlap at a ratio of 22%; however, Jeck et al. demonstrated that only 69 of these circRNAs genes have the same start and end points [10]. The conservation of circRNAs is also manifested in some circRNA exon splice sites amongst different species [19]. Guo et al. revealed that if a certain gene has the ability to encode circRNA in a species, then that gene may also have the encoding ability in other species [20].

## Function of circRNAs

Studies on the function of circRNAs are still limited and challenging. The reported functions of circRNAs are regulating linear RNA transcription, miRNA sponging, protein sponging, binding to proteins, and translating genes into short proteins.

MiRNA sponging is the most widely reported function of circRNAs. Molecular sequences of circRNAs are rich in miRNA response elements (MREs), which can restrict the function of miRNAs by binding to them. This binding in turn releases the target genes that are restricted by miRNA [21]. For instance, the circRNA ZNF91 has multiple miRNA binding sites, including 24 miR-23 binding sites and seven miR-199 binding sites [20]. The circRNA hsa_circ_0012673 is a sponge for miR-22 and releases the inhibition of target gene, human epidermal growth factor receptor 3 (HER3/ErbB3), thereby promoting the proliferation of lung adenocarcinoma cells [22]. Similarly, the testicular-specific circRNA, sex-determining region Y (Sry), has 16 binding sites of miR-38 [23]. Notably, some theoretical papers question the sponging role of circRNAs. They proposed that this crosstalk is impossible since most circRNAs lack or contain only a limited number of miRNA binding sites [24]; thus, the general sponging role of circRNAs continues to be studied. In addition, the lack of clear functions for circRNAs reminds us of a possible fact that only some miRNAs are rich enough in the tissue to truly play a regulatory role in mRNA translation; miRNAs are used more as a buffer to keep translation in equilibrium [25].

Although circRNAs are classified into a noncoding RNA family and usually do not encode proteins, some recent studies have shown the encoding ability of some endogenous circRNAs. Circ-FBXW7, circ-ZNF609, and circMbl were reported to be translatable. The proteins derived from these circular transcripts contain a full protein domain, indicating their functional potential [26]. Yang et al. found that circ-FBXW7 encoded a protein consisting of 185 amino acids, FBXW-185aa, which repressed the tumorigenesis of glioma [27]. Circ-ZNF609 has the ability to bind multiple ribosomes because its main structure contains ‘start’ and ‘stop’ codons, and the transcription is in a cap-independent manner [28].

## The putative effects of circRNAs on the progression of diseases

In recent years, with the widespread application of gene sequencing technologies and bioinformatics methods, an increasing number of circRNAs have been discovered and suggested to be abnormally expressed in various diseases [29]. It was reported that circRNAs were more abundant in the brain than in other tissues, and this result may provide clues to the development of neurological diseases, including Alzheimer’s disease, Parkinson’s disease, and nerve damage [30]. Lei et al. proposed 17 circRNAs, including heart-related circRNA, circRNA-000203, hsa_circ_0082824, and circ-Foxo3, that are positively associated with cardiovascular disease, cardiac fibrosis, myocardial infarction, and atherosclerosis [31].

In the field of tumors, a large number of studies related to circRNAs have been reported in a short time. Hsa_circ_0007534 was more highly expressed in colorectal cancer tissues than in para-cancerous tissues, and the expression of hsa_circ_0007534 was positively correlated with advanced stages of disease and lymph node metastasis. When silenced, hsa_circ_0007534 in the human colon cancer cell lines SW620 and LoVo could inhibit cell proliferation and induce cell apoptosis by regulating the expression of caspase-3, Bcl-2 and Bax, which further suggested the oncogene activity of has_circ_0007534 [32]. Interestingly, it was reported that circRNAs participate in tumorigenesis by acting as miRNA sponges. For example, circC3P1 could sponge to miR-4641, inhibiting proliferation, invasion, and migration of hepatocellular carcinoma via the miR-4641/PCK1 pathway [33]. Overexpression of circNASP in osteosarcoma could relieve its inhibition of FOXF1 by sponging miR-1253 and then promoting cell proliferation, cell cycle progression, and invasion through the circNASP/miR-1253/FOXF1 axis [34]. CicMAN2B2 and hsa_circ_0001649 also participate in the regulation of lung cancer and cholangiocarcinoma, respectively [35,36]. In addition, hsa_circ_0023404 (also known as circRNA_100876), hsa_circ_0013958, hsa_circ_0004277, and hsa_circ_0005075 were found to be biomarkers for non-small cell lung cancer, lung adenocarcinoma, acute myeloid leukemia, and liver cancer, respectively [2,37,38] and might provide new ways for tumor diagnosis and treatment in the future.

## The effects of circRNAs in gynecological diseases

In recent years, there have been increasing reports on the expression and potential biological function of circRNAs in gynecological diseases. The current reports of circRNAs in gynecological diseases are summarized in (Table 1).

**Table 1:** circRNAs that were reported to be associated with gynecological disease

CircRNA alias	Gene	Up/ Down	Disease	Pathway/Regulatory net work	Function/Clinical association	Ref.
mmu_circ_0002861	EGFR	Up	Ovaries	CircEGFR/miR-125a-3p	Promote GCs proliferation	[44]
Chi_circ_0008219	Chr28	Up	Follicle	Sponging chi-miR-34C-5P, chi-miR-483 and chi-miR-1468-3p	Regulate follicular development	[46]
Hsa_circ_103827	HMGCS1	Up	Ovarian aging	Sponging miR-411-5P and miR-625-3P	Cause poor oocytes and embryos quality	[50]
Hsa_circ_104816	IARS	Up	Ovarian aging	Sponging miR-561-5P and miR-140-3p	Suggests ovarian reserve failure and adverse reproductive outcomes	[50]
CircHIPK3 (hsa_circ_000284)	HIPK3 (exons 2)	Up	EOC	–	Associated with lymph node invasion, advanced FIGO, and shorter DFS and OS	[52]
		Up	CC	MiR-506/Snail-2	Promote proliferation and invasion of CC cells	[71]
Hsa_circRNA_070616 Hsa_circRNA_103716	–	Up	-RE	hsa-miR-574-5p-mRNA pathway axis	Regulate embryo implantation	[58]
CircRNA-9119	–	Up	-RE	circRNA-9119/miR26a/ PTGS2	Regulate EECs	[63]
Hsa_circ_0003570	C3	Down	EMT	–	–	[65]
Hsa_circ_0008951	CKS2	Up	EMT	Target miR-29c	Induce a compromised reactivity to progesterone	[65]
			EMT	THRB	Thyroid hormone receptor pathways	[65]
Hsa_circ_0017248	G0S2	Up	EMT	Target miR-145	Inhibit proliferation and invasion	[65]
Hsa_circ_0004712	SCN3B	Up	EMT	Target gene VDR	Cause autophagy, inhibiting apoptosis, and attenuate oxidative stress	[65]
			EMT	Regulate miR-455-3p, miR-876-3p, miR-661 and miR-323a-5p		[66]
Hsa_circ_0002198	ENTPD1	Up	EMT	Regulate miR-455-3p, miR-876-3p, miR-661 and miR-323a-5p		[66]
Hsa_circ_0141539	C1orf116	Up	CC	MiR-518d-5p/519-5p/CBX8	Influence the cell cycle and proliferation, migration and invasion ability of CC cells SiHa and Hela	[68]
Hsa_circ_0031288	PABPN1	–	CC	Bind with HuR	Affect PABPN1 mRNA translation and induce low cell proliferation	[69]
Hsa_circ_0023404	RNF121	Up	CC	miR-136/TFCP2/YAP	Promote CC malignant behavior of proliferation and invasion	[70]
Hsa_circ_0004015	CDK14	Up	CC	Bind to miRNA and regulate gene	Affect cell migration and angiogenesis and radiotherapy resistance	[73]

We used the circBase name (circRNA alias) or other additional names that researchers use for these transcripts.

## Ovarian diseases

Ovaries are important organs for maintaining female hormone balance. Follicles are ovarian functional units, and dysplasia is a common cause of female infertility. Each follicle comprises oocytes surrounded by somatic cells, including granulosa cells (GCs) and theca cells [39]. There are differences in the development of oocytes and the proliferation and differentiation of GCs in adult ovaries and neonates. GCs acquire the ability to secrete or respond to sex hormones such as estrogen, androgen, and progesterone [40]. With the development of RNA sequencing technologies, many circRNAs have been proven to regulate follicular maturity. CircEGFR (circ_0002861, ID: mmu_circ_0002861 in circBase), a 249-nucleotide circRNA that originated from exons 14 and 15 of the EGFR gene, has been widely reported to regulate the cell cycle [41]. Jia et al. found that circEGFR was highly expressed in adult mouse ovaries compared with neonate ovaries and promoted the maturation of follicles. Down-regulating the expression of circEGFR in mice reduced the secretion of estrogen and increased the production of progesterone by GCs, inhibited cell proliferation and promoted apoptosis. These mechanisms might be attributed to the sponging effect on miR-125a-3p, which in turn increased Fyn gene expression and regulated the cell cycle and proliferation. MiR-125a-3p has a variety of biological functions during the process of follicular maturation [42,43], including promoting the transition of cells from the G_1_ phase into the S phase, regulating the expression of cyclin D1, and regulating apoptosis and proliferation. CircEGFR binds to miR-125a-3p, and the function of miR-125a-3p can be repressed by circEGFR, which further increases CYP191 mRNA expression and blocks CYP11A1 expression [44]. In addition, a variety of circular RNAs, including chi_circ_0007167 and chi_circ_0008219, could affect the biological function of follicles. Chi_circ_0008219 contains the binding sites of chi-miR-34c-5p, chi-miR-483, and chi-miR-1468-3P. Chi-miR-34c-5p manipulates embryonic cleavage by interacting with Bcl. The dysregulation of chi-miR-483 is highly and closely related to women with ovulation dysfunction [45]. The expression of chi-miR-1468-3P is differentially expressed in the follicular and luteal phases. Thus, we can easily conclude that chi_circ_0008219 regulates follicular development, ovulation, and embryonic cleavage in goats mainly through the sponging effect of miRNAs [46].

Ovarian aging is a process in which ovarian function gradually diminishes with age and is considered to be the leading cause of age-related female infertility and adverse embryo outcome [47]. Ovarian aging is usually attributed to a gradual decrease in follicular reserve, declined oocyte capacity and poor GCs [48,49]. Cheng et al. found that hsa_circRNA_103827 and hsa_circRNA_104816 were significantly and positively correlated with maternal age and negatively correlated with high quality embryos. The two up-regulated circRNAs have overlapping interacting genes of XIST, KCNQ1OT1, and ANKRD20A9P, which can regulate ovarian aging and abnormal embryonic development. Through these interactions, these two circRNAs manipulate glucose metabolism, mitotic cell cycle, and ovarian steroidogenesis [50].

Ovarian cancer is the leading cause of gynecological cancer mortality [51]. Many circular RNAs have been reported to be abnormally expressed in ovarian cancer and cell lines. CircHIPK3 (hsa_circ_000284), derived from the tumor suppressor gene HIPK3, was reported to be underexpressed in bladder cancer but highly expressed in hepatocellular carcinoma. Liu et al. found that circHIPK3 was highly expressed in epithelial ovarian cancer (EOC) and ovarian cancer A2780, HO-8910, SKOV3, and CAOV3 cell lines. CircHIPK3 regulates the growth of a variety of cancers through sponging miRNAs, amongst which miR-124 is the most famous miRNA [52]

## Embryo implantation

Infertility affects approximately 15% of females in their reproductive age worldwide [53]. Although many women have good embryos, some women experience repeated implantation failure, which may be related to endometrial correlation factors; thus, it is necessary to focus on molecules of endometrial receptivity in patients with repeated implantation failure [54,55]. Qiao et al. found that circRNAs participated in oocyte and preimplantation of embryos [56]. Liu et al. detected multiple differentially expressed circRNAs in repeated implantation failure specimens, in which overexpression of hsa_circRNA_103716 and hsa_circRNA_070616 were found. Both circRNAs could function as sponges of hsa-miR-574-5p. Hsa-miR-574-5p is closely associated with many cancers. Hsa-miR-574-5p can repress the invasion and metastasis of non-small cell lung cancer and colorectal cancer by reducing the expression of MACC-1. In gynecological diseases, overexpression of miR-574-5p can inhibit the growth and metastasis of cervical cancer [57]. More interestingly, Zhang et al. reported that hsa_circRNA_070616 was highly expressed in proliferative endometrium and induced endometrium hyperplasia via miR-574-5p when estrogen was overexposed, which may alter the endometrial receptivity of embryo implantation and cause infertility to a certain extent [58].

Endometrial receptivity is vital for embryo implantation [59], and the acquisition of endometrial receptivity is a complex process [60]. Numerous ncRNAs have been reported to be involved in this progression [61,62]. Zhang et al. detected higher levels of circRNA-9119, prostaglandin-endoperoxide synthase 2 (PTGS2) and lower expression of miR-26a in the receptive endometrium of dairy goats. MiR-26a is widely expressed in endometrial epithelium cells (EECs), and it is the target of P53 and can be regulated by 17β-estradiol (E2) and progesterone (P4). PTGS2 regulated many protein markers in endometrial receptivity, including p-AKT, p-P38, and mTOR. MiR-26a can inhibit the expression of PTGS2 by binding to its 3′ UTR, which further regulates the PTEN, PI3K/AKT/mTOR signaling pathways, P38, and VEGF. In EECs, circRNA-9119 shared the same MRE with miR-26a, exerted as a competing endogenous RNA (ceRNA) and sequestered miR-26a as miRNA sponge, thus protecting PTGS2 by exerting its function [63].

## Endometriosis

Endometriosis (EMT) is a common gynecological benign disease that exhibits malignant-like features, including adhesion, invasion, and angiogenesis. Xu et al. revealed five differential expression circRNAs (circ_0004712, circ_0002198, circ_0008951, circ_0017248, and circ_0003570) in ectopic endometrium (EcE) compared with eutopic endometrium (EuE) by microarray analysis. These differentially expressed circRNAs regulate various biological functions of the endometrium via regulating various genes and miRNAs, such as TP53 and TCF7. Amongst these circRNAs, circ_0008951 has an effect on the endocrine function. The expression level of circ_008951 in the secretory phase is inferior to that in the proliferative phase. Circ_008951 regulates the thyroid hormone receptor pathway, which plays an important role in ovarian EMT. In addition, circ_0008951 targets miR-29c and induces compromised reactivity to progesterone in EMT. Circ_0017248 inhibits cell proliferation and invasion by targetting miR-145 in EMT. Circ_0003570, originating from the MBOAT1 gene, positively regulates PPP1R9A and SOX4 and plays an inhibitory role in manipulating the tumor-like characteristics of ectopic endometrium [64].

This interaction between circRNA and miRNA is not one to one. One circRNA can target multiple miRNAs, and one miRNA can be regulated by more than one circRNA; for example, miR-503, which causes cell apoptosis, blocks the cell cycle, and inhibits cell proliferation and angiogenesis, is regulated by both circ_0004712 and circ_0002198 [65]. Circ_0004712 (DGKG) and circ_0002198 have many overlapping biological and functional interactions, and both circRNAs regulate the abnormal expression of four microRNAs (miR-455-3p, miR-876-3p, miR-661, and miR-323a-5p) and two genes (SCN3B and ENTPD1). Amongst these miRNAs, miR-876 induces cell apoptosis, and miR-66a activates p53 and inhibits cell cycle progression. By targetting these miRNAs, circ_0004712 and circ_0002198 block endometrial cell proliferation and induce apoptosis via MER/ERK and c-myc. The SCN3B gene has an effect on the p53-dependent apoptotic pathway, and ENTPD1 can regulate T cells [66].

## Cervical cancer

Cervical cancer (CC) is the most common gynecological malignancy [67]. Various circRNAs are abnormally expressed in CC tissues and cell lines. Hsa_circ_0141539 is produced by the exon of the Clorf116 gene, which is also known as circRNA8924. Hsa_circ_0141539 is highly expressed in CC tissues in addition, the expression level of hsa_circ_0141539 was positively correlated with tumor size, FIGO stage, and myometrial invasion. Hsa_circ_0141539 exerts oncogenic activity by regulating the cell cycle, promoting cell transformation from the G_0_/G_1_ phase to the S phase, increasing the proportion of cells in the S phase, increasing the expression of cyclin D1, and reducing the expression of apoptosis-related proteins, such as Bax. Hsa_circ_0141539 competitive binds to miR-518d-5p/519-5p, releasing its restraint to the downstream genes and promoting the expression of its target gene, CBX8, and promoting migration and invasion and affecting the cell cycle in the CC cell lines SiHa and HeLa. As the core component of the PRC1 complex, CBX8 is the initiation factor of tumor progression and is closely related to tumorigenesis, invasion, migration, and prognosis [68]. In HeLa cells, HuR, an extensively studied RBP, binds to multiple circRNAs without affecting their expression abundance. Amongst these circRNAs, hsa_circ_0031288, also known as circPABPN1 and originating from PABPN1 pre-mRNA, is targetted by HuR. A small amount of circPABPN1 can seize plentiful protein HuR from target mRNA [69]. The HuR-circPABPN1 complex affects the function of HuR, preventing HuR from binding to its homologous PABPN1 mRNA, limiting the expression of PABPN1 mRNA, and consequently lowering the translation of PABPN1 and lowering cell proliferation [69]. However, circPABPN1 does not affect the ability of HuR to interact with other mRNAs.

Hsa_circ_0023404, derived from the RNF121 gene on chromosome 11, and circRNA_000284 are up-regulated in CC tissues and cell lines. Hsa_circ_0023404 regulates CC via YAP signaling in a TFCP2-dependent manner. Hsa_circ_0023404 activates the YAP signaling pathway via sponging on miR-136. MiR-136 expression is negatively correlated with hsa_circ_0023404, and it targets TFCP2; moreover, TFCP2 activates the YAP signaling pathway. In summary, hsa_circ_0023404 plays a pivotal role in CC, promoting the expression of TFCP2 by sponging miR-136, consequently activating the YAP signaling pathway and thus contributing to CC proliferation and invasion. Hsa_circ_0023404/miR-136/TFCP2/YAP is a new axis loop that regulates the malignant behavior of CC [70]. A similar mechanism also occurs with hsa_circ_000284. Hsa_circ_000284 is derived from exon 2 of the HIPK3 gene, is synthesized from direct splicing and is stably expressed in tissues and cell lines. A tissue microarray assay revealed that circRNA-000284 was significantly up-regulated in CC tissues. Knockdown of circRNA_000284 inhibits cell proliferation and invasion and causes cell cycle arrest in the G_0_/G_1_ phase. Hsa_circ_000284 shares a complementary sequence with miR-506 and can sponge on miR-506. Snail-2 is a target of miR-506 and is positively regulated by circRNA-000284. The circRNA_000284/miR-506/Snail-2 network targets miR-506 via Snail-2, which regulates the proliferation and invasion of CC [71].

In recent years, with the improvement of radiotherapy technology, postoperative adjuvant therapy has become a routine treatment for CC patients, but radiotherapy resistance is still the cause of poor efficacy of *in situ* high-grade CC [72]. Yu et al. divided HeLa cells into irradiated and sham-irradiated groups. A total of 153 circRNAs were detected as abnormally expressed by high-throughput sequencing; of these circRNAs, 76 circRNAs were up-regulated and 77 were down-regulated. The top up-regulated and down-regulated circRNAs were used for further circRNA-miRNA-target gene interaction network analysis, resulting in the discovery that one single circRNA can target multiple miRNAs. For instance, hsa_circ_0004015, which was highly expressed in the irradiated group, binds to miR-3163, miR-3065-5p, miR-551b-5p, miR-4311, and miR-875-3p. In turn, these miRNAs affect the expression of approximately 380 target genes, including ZYX, PRKAR1A, BTAF1, LRP3, and ETS1, which promote cell migration and angiogenesis. These circRNA-miRNA-gene interaction nets may help provide a better understanding of radiotherapy resistance and provide tumor response to radiation [73]

## The roles of circRNAs in gynecological diseases

The ovary is the main organ that secretes sex hormones and are also an important place to produce and discharge ootid. Follicles are the basic functional unit of oocyte genesis and development. During the development of follicles, the maturation and proliferation of follicles are regulated by circRNAs, including circEGFR, chi_circ_0007167, and chi_circ_0008219. CircEGFR induces an increasing release of estrogen and decreasing release of progesterone in GCs. The imbalance of estrogen and progesterone has important impacts on female endometrial carcinoma and breast tumors. Thus, we can infer that circEGFR has a great effect on those tumors. The up-regulated circRNA_103827 and circRNA_104816 are related to ovarian senescence, and they can induce deterioration of the follicular microenvironment, with the direct consequence of poor quality of oocytes and embryos quality. These circRNAs can be used to predict in intro fertilization prognosis, ovarian reserve failure, and adverse reproductive outcomes and can be applied to the management of female infertility and some reproductive therapies. In addition to participating in the physiological processes of the ovary, some circRNAs have also been shown to be associated with the pathogenesis of ovarian tumors. For example, circHIPK3 can be used as a biomarker of EOC, and its expression level is associated with poor tumor prognosis, lymph node invasion, and advanced FIGO stage; moreover, circHIPK3 independently predicts a shorter disease-free survival (DFS) and overall survival (OS) time of EOC patients.

Endometrial receptivity is a comprehensive state of endometrium receiving embryo implantation and is also a key factor for successful embryo implantation. Estrogen can alter endometrial receptivity by regulating hsa_circRNA_103761 and hsa_circRNA_070616. CircRNA-9119 manipulates the growth of endometrial epithelia cells by forming circRNA-9119/miR26a/PTGS2 pathways. MiR-26a regulates endometrium cell proliferation and cell survival through the regulation of the cell cycle and cell survival factors. Therefore, these circRNAs can be used as potential biomarkers for estrogen overexposure and endometrial receptivity abnormalities, offering new candidates for the molecular diagnosis and clinical treatment of embryo implantation failures.

In EMT, the abnormally expressed circRNAs target miRNAs and genes, which not only regulates endocrine reactivity of the endometrium but also affects the tumor-like characteristics, cell proliferation, apoptosis, and vascular formation of the endometrium. The present study revealed the potential application of circRNAs in EMT. CircRNAs are candidate factors for activating EMT and are promising diagnostic markers and treatment targets.

**Figure 1:**
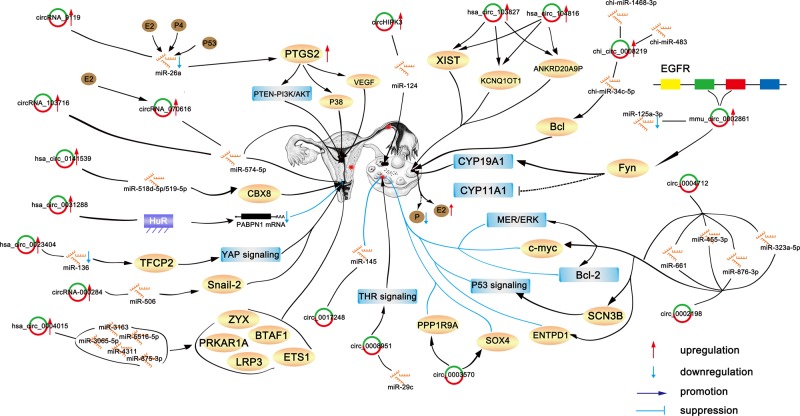
Function and interaction of circRNAs in gynecological disease CircRNA-mediated mechanisms in gynecological disease. A circRNA exerts its function mainly by binding to miRNA and targetting corresponding genes and pathways.

The discovery of circRNAs provides a new theoretical foundation for the development of CC and opens up a new direction for targetted therapy. Many circRNAs have been shown to be abnormally expressed in CC. CircRNAs have roles as oncogenes in CC and promote the proliferation, growth, and migration of this tumor. The way circRNAs function is mainly by competitive binding to miRNAs. For example, hsa_circ_0141539 competitively binds to the miR-518d-5p/519-5p family, and hsa_circ_0023404 plays a carcinogenic effect on CC by targetting the miR-136/TFCP2/YAP pathway axis. CircRNAs also bind to RBPs and selectively inhibit the post-transcriptional expression of the corresponding homologous pre-mRNA. Binding of HuR to circPABPN1 inhibits the binding of HuR to its homologous mRNA, PABPN1, resulting in a decrease in PABPN1 translation and lower cell proliferation; however, circPABPN1 does not affect the binding of HuR to other mRNAs. Some circRNAs, including circRNA_103716, circRNA_070616, and hsa_circ_0031288, have been shown to inhibit the proliferation and metastasis of CC cells. There is a complex relationship between various novel circRNAs and carcinogenesis signaling pathways (Figure 1). Therefore, we may infer that circRNA is a new biomarker and potential therapeutic target for CC. Targetting circRNA may be the future direction of the development of new therapeutic strategies.

## Regulatory strategies targetting circRNAs

Although many functions of circRNA have not been thoroughly revealed, it has been proven to play a role in multiple pathways of disease. The regulation of circRNA on genes involves pre- and post-transcriptional regulation; thus, the regulation of circRNA, a central link factor, is of vital importance. The unique cyclic structure lacks the free ends necessary for exonuclease-mediated degradation, making them resistant to a variety of RNA degradation mechanisms. Compared with linear mRNA, circRNAs have a longer lifespan and have a broader application prospect [74]. To further understand the function of circRNA *in vivo*, it is necessary to construct circRNA *in vitro* and stabilize its expression after loading into cells to regulate circRNA. Many studies have proposed effective methods to regulate circRNA expression in cells for identifying more circRNA functions in future studies [75]. In the design of circRNA primers, due to the ring-specific structure formed by its reverse splicing, primers must span the bases around the ring-connecting sites. CircPrimer is software that can be used by beginners to search for and extract base sequences and to design probes containing the connecting sites for specific detection [76]. Alexander Wesselhoeft et al. proposed an engineered permuted group 1 catalytic intron-based system for exogenously synthesized circRNA, which can exogenously synthesize circRNA with a base sequence of up to 5 kb and can be inserted into different coding regions. Then, the synthesized circRNA can be efficiently introduced into eukaryotic cells by a cationic lipid transfection reagent and is capable of efficient and sustained protein expression in eukaryotic cells. These methods are very effective in regulating the expression levels of circRNAs [77].

Strategies for targetted silencing of circRNAs are mainly RNA interference that is complementary to the targetted mRNA sequence, and RNAs are guided to be cleaved by the RNA-induced silencing complex. Degradation of RNA templates eventually leads to silencing genes and circRNAs and inhibiting protein translation [78]. Targetted silencing can also be combined with CRISPR/Cas9 technology to knock out circRNAs of interest by knocking out the repeated elements of the flanking introns of circRNAs. The Cas9 protein is an enzyme that cuts specific sites of the genome under the guidance of guide RNA (gRNA). In this process, once the dsDNA is cut by Cas9, the cell will start the DNA repair mode, which will cause gene deletion and insertion. Through the design and control of gRNA and Cas9 enzymes, knockout of genes can be achieved [79]. Cas9 can be used to knock out the circRNA gene of interest, remove the side intron repeat element to reduce its expression, while not affecting the linear RNA production of the host gene and maintaining the integrity of the host gene. The regulation of circRNA expression levels is conducive to further exploring its functions.

## Future perspective

Notably, multiple tumors have demonstrated that genes can be rearranged to produce fusion genes and fusion circRNAs (f-circRNAs) [80]. It is a new way to promote tumors. Usually, the repeated complex sequences within eukaryotic introns will lead to the fusion of two different genes by way of chromosomal displacement. If the complementary repeated complex sequences are generated from the upstream and downstream regions of the fused intron region that are close enough, reverse shear may occur to form a f-circRNA. For example, f-circPR is produced by the fusion of the PML-RARA gene in promyelocytes, f-circm9 is produced by the fusion of the MLL-AF9 gene in acute myeloid leukemia, and EWSR1-FLI1 in Ewing’s sarcoma and EML4-ALK1 in lung cancer have all been reported to produce one or more fusion circRNAs after gene fusion [81]. F-circRNA contributes to cell transformation, promotes cell viability, and post-treatment drug resistance, and has tumor-promoting properties *in vivo* models [82]. F-circRNA is another mechanism by which genes promote tumors through rearrangement, which can increase the cell proliferation rate and clonability and cause loss of contact inhibition in cells [83]. Although f-circRNA has been proven by many research groups in multiple experiments, the specific mechanism of its role is still unclear, and more *in vivo* and *in vitro* experiments are needed to explore the pathways and mechanisms involved in the regulation of f-circRNAs to further and more comprehensively understand them and their biological functions.

Although a large number of reports have suggested that a circRNA can be used as a specific tumor marker, a single circRNA often needs to be combined with other markers or act on downstream related factors to regulate the tumor more effectively. Many studies have shown that a circRNA may act on a certain miRNA, but miRNA can play a role in a variety of tumor pathways, thus reducing the specific molecular markers of circRNA in tumor prediction. Therefore, it is likely that circRNAs are common driving mechanisms of many tumor-related signaling pathways or common tumor-driven byproducts or final products. Therefore, further understanding of miRNAs, proteins and pathways that interact with circRNAs is required. The localization of the specific role of circRNAs in tumors can clarify the most important biological functions of circRNAs.

Although the miRNA sponge effect is an important function of circRNA, some circRNAs do not contain perfect trapping sites for miRNAs; therefore, we should also pay close attention to other ways that circRNAs function besides the sponging effect [75]. For example, although circRNAs were initially classified as noncoding RNAs, some experiments have shown that circRNA sequences contain an internal ribosomal entry site-driven open reading frame (ORF) that can translate functional proteins, and these short peptide proteins can regulate cell proliferation and tumorigenesis [27,84]. The encoded protein plays an important role in the function of a circRNA, but the similarities and differences between this kind of short peptide protein and common functional protein still need to be further studied.

In addition, there are still many problems to be studied, which need to be solved one by one in the future. As recently shown, exogenous circRNA transfection can activate cells to produce antiviral gene products such as OAS, PKR, and RIG-I [85]. To inhibit the immune response of the body to circRNAs, further adjustments of circRNAs and nucleotide design, such as nucleotide modification to reduce the immunogenicity generated by the introduction of circRNA into the body, should be considered in experiments. Despite the doubts in the circRNA studies, the importance of circRNAs in the diagnosis and treatment of diseases cannot be denied. Since the diagnosis of the disease depends on the clinical characteristics and biological phenotypes of the patient, the combination of circRNAs and other biomarkers will greatly contribute to the diagnosis and treatment of tumors.

**Figure 2:**
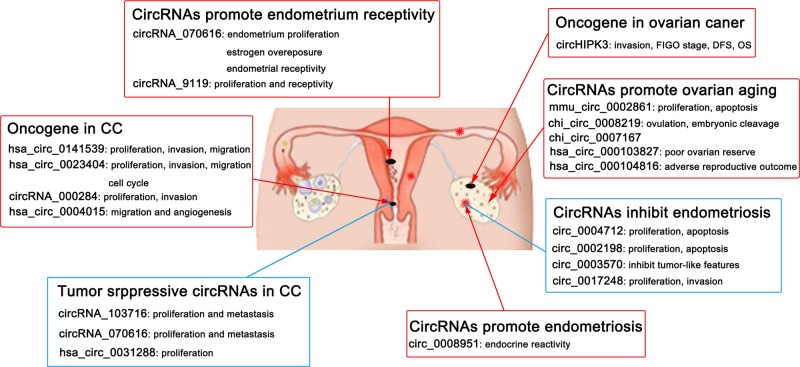
Biomarkers and diagnostic role of circRNA in gynecological diseases CircRNAs promote and inhibit gynecological diseases. Emerging evidence has confirmed the dysregulation of circRNAs in gynecological diseases. Thus, circRNAs may be a potential therapeutic target for gynecological diseases and may be potential biomarkers for diagnosis and prognosis.

Collectively, these studies have deepened our understanding of circRNAs and further demonstrate the complexity of the molecular mechanisms involved in disease. As mentioned above, many circRNAs are considered to be potential diagnostic and therapeutic markers of disease and are involved in the physiological and pathological processes of gynecological diseases; in addition, the study of circRNAs provides new possibilities for the diagnosis and treatment of diseases (Figure 2). Studies of circRNAs in gynecological tumors mainly focus on ovarian cancer, EMT and CC, and more studies are needed for other gynecological diseases, such as endometrial carcinoma, choriocarcinoma, vulvar squamous cell carcinoma, uterine sarcoma, and malignant teratoma. With the application of high-throughput RNA sequencing and data analysis methods, the roles of circRNAs in gynecological diseases will be further explored and will continue to be enriched and improved with further research. The understanding of circRNAs related to gynecological diseases will undoubtedly provide a useful introduction and valuable new hypothesis for diseases, eventually achieving a major breakthrough for clinical applications.

## Abbreviations

CC: cervical cancer
circRNA: circular RNA
DCC: deleted in colorectal carcinoma
DFS: disease-free survival
EEC: endometrial epithelium cell
EMT: endometriosis
EOC: epithelial ovarian cancer
f-circRNA: fusion-circRNA
FIGO: international federation of gynecology and obstetrics
GC: granulosa cell
gRNA: guide RNA
HuR: human antigen R
MRE: miRNA response element
ORF: open reading frame
OS: overall survival
PTGS2: prostaglandin-endoperoxide synthase 2
RBP: RNA binding protein
tRNA: transfer RNA
YAP: yes associated protein
